# Compound design of a patient-derived 3D cell culture system modelling early peritoneal endometriosis

**DOI:** 10.1242/dmm.052436

**Published:** 2026-02-02

**Authors:** Muhammad D. R. Rahmana, Christopher J. Hill, Bettina Wilm, Dharani K. Hapangama

**Affiliations:** ^1^ Department of Women's and Children's Health, Institute of Life Course and Medical Sciences, University of Liverpool, Liverpool L7 8TX, UK; ^2^ Liverpool Women's Hospital NHS Foundation Trust, Member of Liverpool Health Partners, Liverpool L8 7SS, UK

**Keywords:** 3D culture, Patient-derived, Peritoneum, Endometrium, Peritoneal endometriosis, Superficial endometriosis

## Abstract

Peritoneal endometriosis causes pelvic pain and infertility, but the underlying mechanisms related to these symptoms are not fully understood. Endometriosis diagnosis is typically delayed; thus, patient samples are unsuitable to study early endometriosis formation *in situ*. We generated a 3D co-culture model of early peritoneal endometriosis using patient-derived primary cells, providing unique opportunities to examine endometriotic lesion initiation and progression. The successful assembly of a simple peritoneum layer model comprising a mesothelial monolayer, basement membrane and underlying fibroblasts was achieved by embedding human peritoneal fibroblasts in a Matrigel-collagen I matrix and subsequent seeding with a layer of donor-matched human peritoneal mesothelial cells, while secretion of tissue plasminogen activator demonstrated functional mesothelial physiology. Endometrial epithelial organoids were co-cultured with endometrial stromal cells to form endometrial assembloids mimicking shed endometrial tissue fragments at menstruation, which adhered onto the peritoneal layer model, simulating early endometriotic lesion formation. Our modifiable superficial endometriosis model allows for further refinement to determine the underlying molecular mechanism(s) involved in endometriotic lesion formation.

## Teaser

**Summary:** Endometriosis cannot be accurately modelled in laboratory animals. Our *in vitro* peritoneal endometriosis model uses patient-derived cells to accelerate understanding of disease onset processes, expediting the development of novel treatment options.

## INTRODUCTION

Endometriosis is a common, incurable, chronic inflammatory condition associated with significant morbidity in at least one in ten women of reproductive age, equating to over 190 million women globally (Horne and Missmer, 2022; Rahmioglu et al., 2023). It typically causes debilitating chronic pelvic pain and is associated with subfertility, affecting the quality of life and wellbeing of patients, with an annual cost estimated at over £8.2 billion to the UK economy attributed to productivity losses and healthcare expenses (Saunders and Horne, 2021). The available treatments mainly consist of hormones, which are contraceptive and associated with significant side effects, while surgical excision is risky and not curative. More effective and fertility-preserving treatments are thus urgently needed. Understanding endometriosis pathophysiology and identifying key molecular markers as targets for therapeutic interventions are essential for the development of novel curative treatments (Sourial et al., 2014).

Traditionally, endometriosis is defined as the presence of abnormally located endometrium-like epithelial glands and stromal tissue outside of the uterus. There are three main types – superficial peritoneal, ovarian and deep endometriosis. Superficial peritoneal endometriosis, in which endometriosis lesions are located on the peritoneal surface of the pelvis, is the most common variety requiring an invasive surgical procedure (laparoscopy) for diagnosis, which is typically delayed (Giudice et al., 2023). It is also the most challenging to manage owing to high recurrence rates (>70%) even after surgical excision (Nisolle and Donnez, 2019). The most accepted theory of endometriosis initiation is ‘Sampson's theory’, which proposes that sloughed endometrial tissue at the time of menstruation is transferred to the pelvic cavity via trans-tubal retrograde flow, where it attaches, invades and grows on the peritoneal surface, while retaining an endometrial-like phenotype (Hapangama et al., 2012; Young et al., 2013). The peritoneal layer is thus postulated to play an important role in this process.

The peritoneum is composed of a simple epithelial mesothelium, basement membrane and underlying vascularised submesothelial stroma containing fibroblasts and other cell types (Herrick and Wilm, 2021). The peritoneum acts not only as a protective barrier but also interacts with macrophages within the cavity (Buechler et al., 2019) and has an important role in fluid transport (Mutsaers et al., 2016). In response to endometriosis, peritoneal dialysis or surgical injury, the peritoneum can undergo pathophysiological changes leading to scarring. Both endometriosis and metastatic peritoneal disease involve the invasion of cells into the peritoneal layer, suggesting common cellular and molecular mechanisms that modulate mesothelial and submesothelial cell behaviour (Li and Guo, 2022; Young et al., 2013). However, detailed analyses of the mechanisms regulating cell attachment, invasion and scar formation in the peritoneal layer are lacking in the published literature.

Endometriosis tissues retain hormone responsiveness and undergo cyclical growth, differentiation, bleeding and regeneration, incurring an inflammatory response and scarring at the ectopic sites (Drury et al., 2018; Mathew et al., 2016; Sourial et al., 2014). The associated chronic pain symptoms are particularly exacerbated at the time of menstruation, and endometriosis is directly relevant to the menstruation process, which only occurs in women and upper-order primates (Sourial et al., 2014).

As menstruation is absent in most laboratory animals, it cannot be accurately modelled *in vivo*. Complex *in vitro* modelling using patient-derived materials is thus essential to understand endometriosis aetiology and pathophysiology. The existing *in vitro* models of peritoneal endometriosis lack inclusion of endometrial and peritoneal cell types (Prechapanich et al., 2014; Song et al., 2023), have no 3D configuration (Cho et al., 2023; Yu et al., 2015) and do not use patient-derived cells (Miller et al., 2017; Pluchino et al., 2019; Slayden et al., 2023); thus, they are poor mimics of the lesions in patients.

Therefore, to overcome these deficiencies, we developed a 3D multicellular patient-derived model of early peritoneal endometriosis, containing the main endometrial and peritoneal cell types.

## RESULTS

### Isolated primary peritoneal mesothelial cells and fibroblasts demonstrate cell type-specific characteristics

**Fig. 1.**
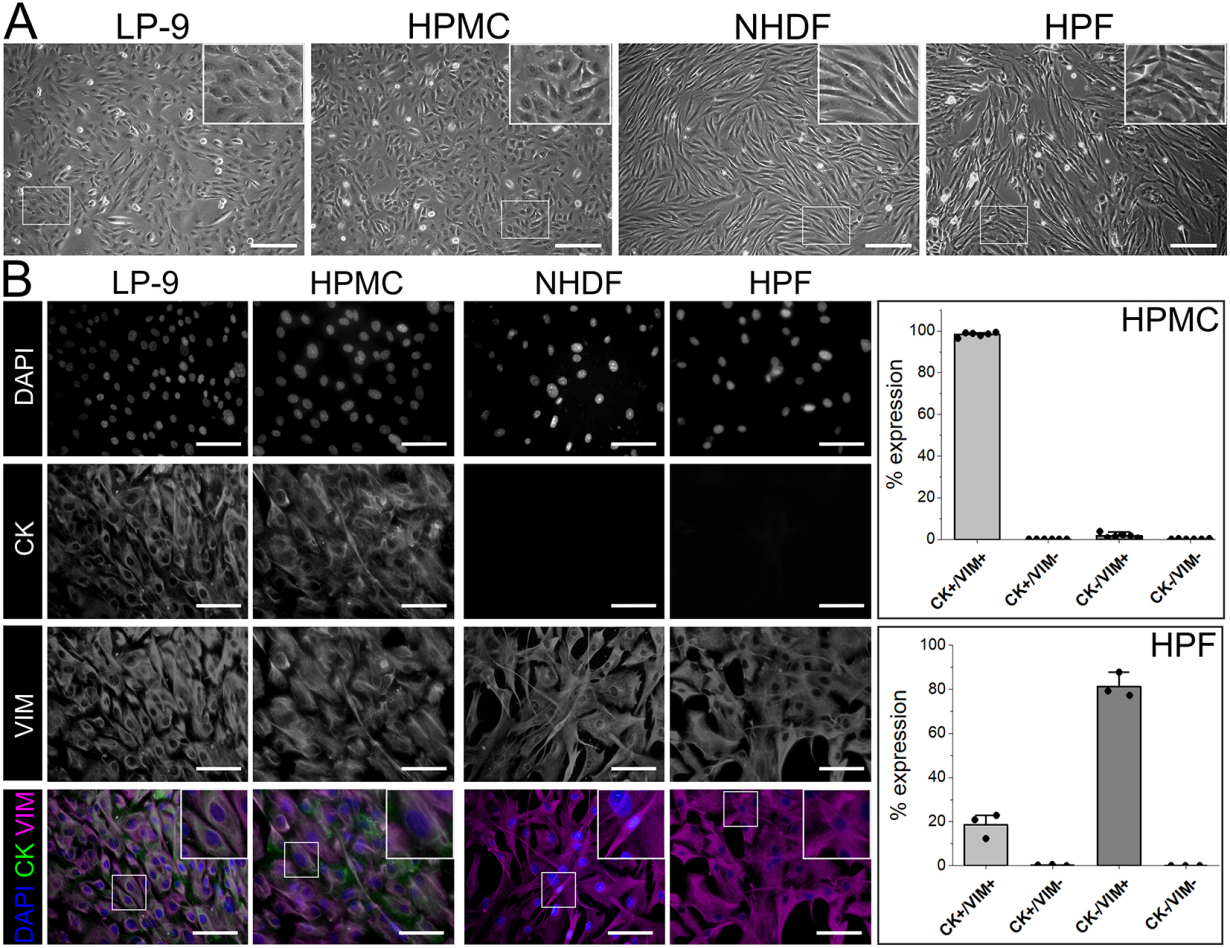
**Characterisation of primary human peritoneal mesothelial cells (HPMCs) and human peritoneal fibroblasts (HPFs) compared to the LP-9 mesothelial cell line and normal human dermal fibroblasts (NHDFs).** (A) Representative phase-contrast micrographs of LP-9, HPMCs, NHDFs and HPFs. Mesothelial and fibroblast cells exhibit cobblestone and spindle-like morphologies, respectively. (B) We observed localised expression of the cytoskeletal markers cytokeratin (CK) and vimentin (VIM) in LP-9 cells and HPMCs, while VIM, but not CK, was expressed in NHDFs and HPFs. HPMCs (*n*=6) showed a high percentage of CK^+^/VIM^+^ cells [98.20±1.05% (mean±s.d.)], while HPFs (*n*=3) exhibited 81.36±5.63% CK^−^/VIM^+^ cells. Scale bars: 200 µm (A); 50 µm (B).

Peritoneal wash-derived human peritoneal mesothelial cells (HPMCs) and fallopian tube mesentery-derived human peritoneal fibroblasts (HPFs) were initially characterised by phase-contrast microscopy to distinguish their morphology. Morphological characteristics of HPMCs and HPFs were compared to the human mesothelial cell line LP-9 and primary normal human dermal fibroblasts (NHDFs), respectively, in a 2D cell culture system. At passage (p)2, HPMCs had a cobblestone appearance comparable to that of LP-9 cells at p7, demonstrating their expected mesothelial morphology. HPFs at p1 exhibited a spindle-shape appearance comparable to that of NHDFs at p11 (Fig. 1A). To confirm peritoneal mesothelial and fibroblast cell phenotypes, we performed dual immunofluorescence staining of cytoskeletal markers cytokeratin (CK; also known as KRT; expressed in epithelial cells) and vimentin (VIM; expressed in mesenchymal cells) (Dauleh et al., 2016) (Fig. 1B). Our findings indicate that CK and VIM were localised in the cytoplasm of the peritoneal mesothelial cells as previously described (LaRocca and Rheinwald, 1984), while the NHDFs and HPFs expressed VIM but lacked CK. Furthermore, scoring for the ratio of CK and VIM expression in six biological HPMC samples showed that the HPMCs at p2 had a very high percentage of CK^+^/VIM^+^ cells [98.20±1.05% (mean±s.d.)]. Meanwhile, isolated HPFs at p2 from three biological samples exhibited CK^−^/VIM^+^ expression in 81.36±5.63% cells, indicating their fibroblast characteristics. However, HPFs also contained 18.64±5.63% CK^+^/VIM^+^ cells, suggesting contamination with mesothelial cells (Fig. 1B).

HPMCs and HPFs were further characterised at p2 using established mesothelial markers including Wilms tumour protein 1 (WT1), mesothelin (MSLN) and podoplanin (PDPN; which also detects lymphatic cells) (Herrick and Wilm, 2021); the epithelial tight junctional marker zonula occludens 1 (ZO1); the stromal/mesenchymal cell marker CD90; and smooth muscle cell markers α-smooth muscle actin (αSMA; also known as ACTA2) and calponin (CPN), based on the CellMarker database. We also included peritoneal fibroblast marker fibroblast specific protein 1 (FSP1) (Witowski et al., 2015) and tumor endothelial marker 1 (TEM1), which we identified as highly abundant in fibroblast cells of the gastrointestinal tract using the Human Protein Atlas database. Our analysis demonstrated mesothelial identity in HPMCs by nuclear WT1 and perinuclear MSLN expression (Fig. S1A). Epithelial characteristics of isolated HPMCs were confirmed by uniform expression of ZO1 at the cell membrane (Fig. S1A). Characteristics of HPFs were supported by the expression of TEM1 on the cell surface, and the prominent expression of αSMA and CPN as stress fibres in the cytoplasm (Fig. S1B). However, we also detected low levels of PDPN expression in these cells (Fig. S1B).

### Generating a composite 3D hydrogel construct using patient-derived peritoneal cells *in vitro*

**Fig. 2.**
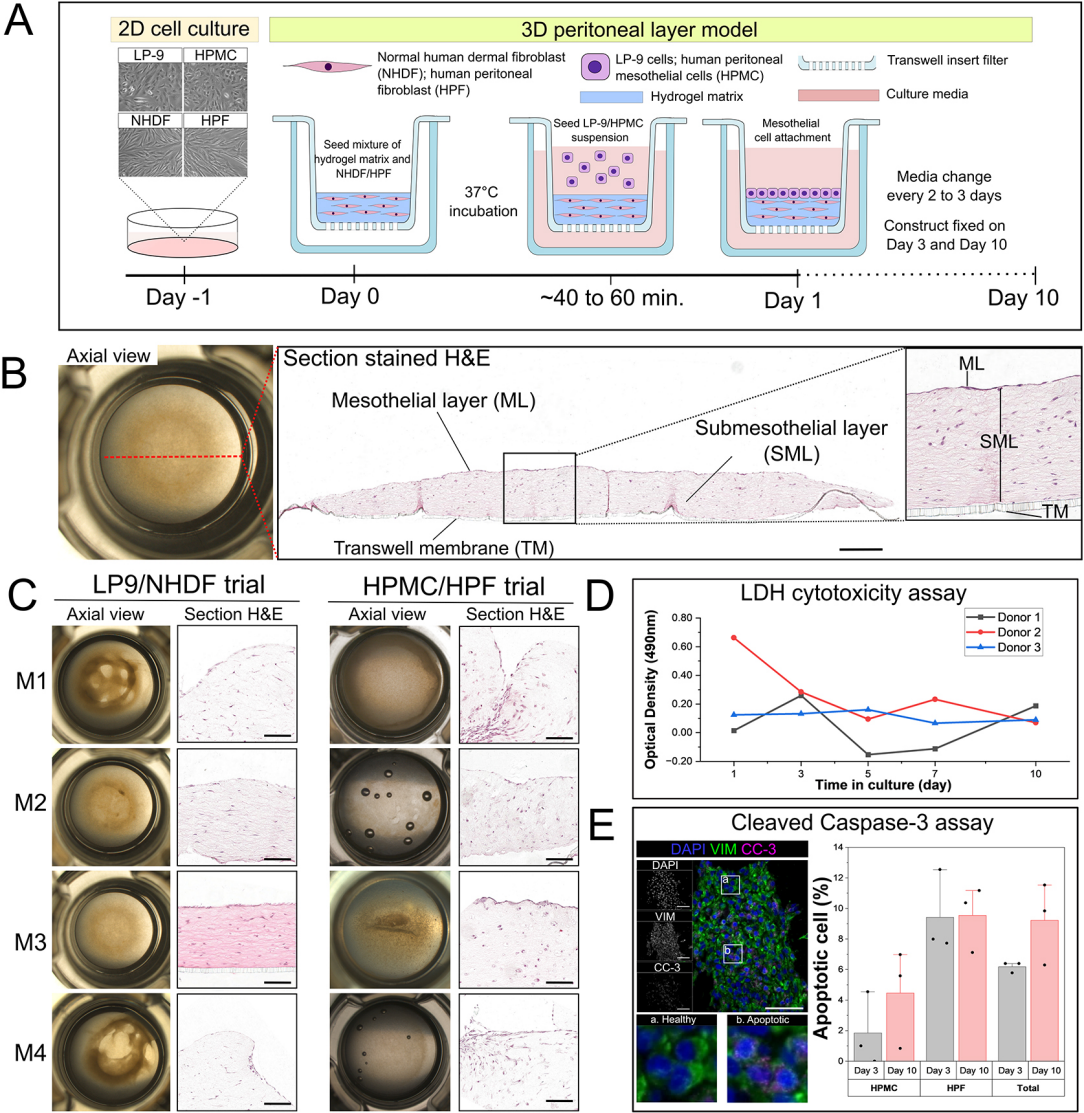
**Establishing composite 3D hydrogel constructs using peritoneal mesothelial cells and fibroblasts.** (A) Schematic illustration of model construction and culture timeline. (B) Representative axial view [also seen in C (M3)] and Haematoxylin and Eosin (H&E)-stained section of a hydrogel construct showing the formation of a mesothelial monolayer (ML) and submesothelial layer (SML) on a transwell membrane (TM). (C) Representative images of hydrogel matrices using M1 (collagen I), M2 (70:30 collagen I:Matrigel ratio), M3 (50:50 collagen I:Matrigel ratio) and M4 (collagen I+human fibronectin). Construct generated with matrix combination M3 demonstrated minimal contraction in LP-9/NHDF and HPMC/HPF trials. (D) Lactate dehydrogenase (LDH) cytotoxicity assay in M3 composite hydrogel constructs containing HPMC/HPF (*n*=3 donors) over a 10-day culture period. (E) Dual immunofluorescence staining of cleaved caspase-3 (CC-3) and VIM to detect apoptotic HPMCs/HPFs in M3 constructs on day 3 and day 10 of culture (*n*=3). Scale bars: 300 µm (B); 100 µm (C); 50 µm (E).

An *in vitro* peritoneal layer model was generated by incorporating peritoneal mesothelial cells and fibroblasts in a 3D co-culture system (Fig. 2A). Initial experiments were performed using commercially available cells (LP-9 and NHDFs) before trialling patient-derived cells (HPMCs and HPFs). A hydrogel-fibroblast (NHDFs or HPFs)-mixture mimicking the submesothelial layer was placed in the upper compartment of a transwell insert. This design allowed sufficient access to nutrients and supported the matrix to remain flat (Estermann et al., 2023). Mesothelial cells (LP-9 or HPMCs) were then seeded onto the surface of the hydrogel-cell matrix to mimic the mesothelial layer. In our approach, we used HPMCs and HPFs from the same patients to ensure compatibility of the cells within the constructs (Table S1). We cultured the composite hydrogel constructs for up to 10 days and analysed the histological structure of the constructs on days 3 and 10. Axial observation of composite hydrogel constructs revealed the formation of flat-shaped disks *in vitro* (Fig. 2B). Histological analysis revealed that a single-cell layer covered the surface of the hydrogel-cell matrix, suggesting the formation of a mesothelial layer by day 3. The hydrogel matrix contained embedded cells mimicking the submesothelial stroma (Fig. 2B). To determine the optimal composition of the hydrogel scaffold, four different combinations were trialled: M1 (collagen I), M2 (70:30 collagen I:Matrigel), M3 (50:50 collagen I:Matrigel) and M4 (collagen I+human fibronectin). Composite hydrogel cultures containing LP-9/NHDF or HMPC/HPF combinations demonstrated differing levels of contraction on day 3 post-formation (Fig. 2C). Histological analysis of transverse sections indicated that different matrix compositions affected the structural appearance of the hydrogel constructs; the upper contour of the constructs varied between matrix combinations. However, matrix combination M3 provided the best support in establishing an *in vitro* model of the peritoneal layer with minimal contraction using both commercial cells (LP-9 and NHDFs) and patient-derived cells (HPMCs and HPFs). Therefore, matrix combination M3, consisting of equal parts collagen I and Matrigel, was taken forward. Furthermore, matrix combination M3 had similar submesothelial thickness to that of biopsies (Fig. S2). The average submesothelial thickness of the LP-9/NHDF constructs using matrix combination M3 was 313.97±72.58 µm (*n*=3), while in HPMCs/HPFs constructs it was 341.16±112.08 µm (Table S2). This finding indicates that the submesothelial layer of our 3D peritoneal model was within the range of the submesothelial dimensions (300-500 µm) of a healthy adult (Schaefer et al., 2016).

To assess cell viability within the composite hydrogel over the experimental period, conditioned media from the basolateral and apical compartments of construct-containing transwell inserts were subjected to lactate dehydrogenase (LDH) cytotoxicity analysis. LDH release remained relatively static across 10 days of culture in constructs composed of cells from three donors (Fig. 2D). A high level of LDH release was observed on day 1 of culture in donor 2, likely due to cell damage induced following model generation. However, LDH release was comparable across donors by day 3. The number of apoptotic cells within the constructs was quantified by cleaved caspase-3 expression (Fig. 2E). HPMC and HPF cells within the constructs were highly viable in the hydrogel constructs, with overall 5.92±0.42%, and 9.22±2.67% of apoptotic cells found at 3 and 10 days of culture, respectively. These findings suggest that the composite 3D hydrogel construct is suitable for long-term culture of patient-derived peritoneal cells.

### The composite 3D hydrogel construct is a simple mimic of the peritoneal layer in architecture and function

**Fig. 3.**
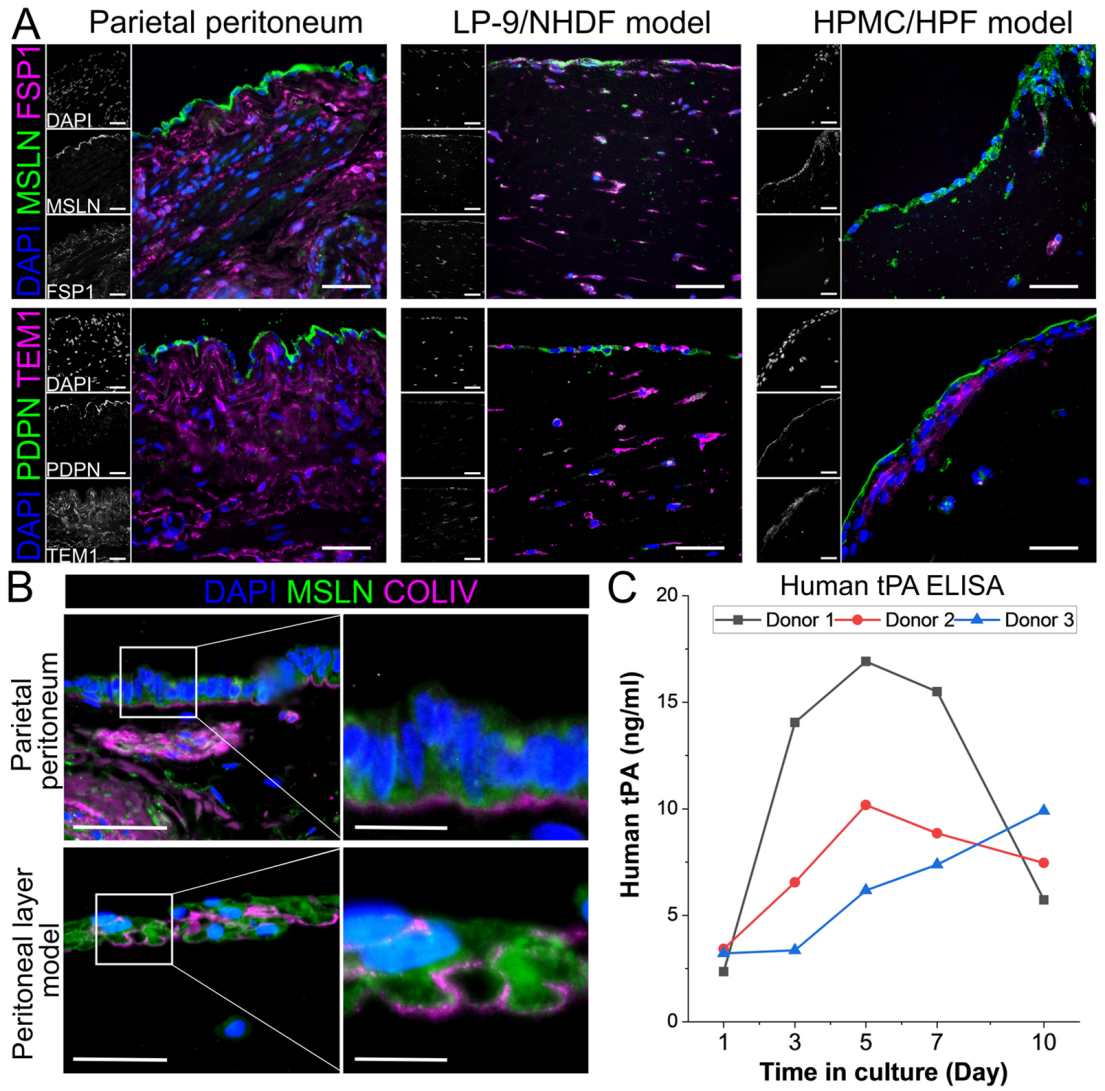
**Histological and functional analysis of the human parietal peritoneum and peritoneal layer models.** (A) Histological staining of transverse sections through parietal peritoneum and composite 3D hydrogel constructs composed of LP-9/NHDFs and HPMCs/HPFs. Immunofluorescence using antibodies against the mesothelial markers podoplanin (PDPN) and mesothelin (MSLN), and submesothelial markers fibroblast specific protein 1 (FSP1) and tumor endothelial marker 1 (TEM1). (B) Colocalisation of MSLN and collagen IV (COLIV) suggesting spontaneous basal lamina formation. (C) Human tissue plasminogen activator (tPA) enzyme-linked immunosorbent assay (ELISA) to determine the functionality of the mesothelial cells in models assembled with HPMCs from three different donors over a 10-day culture period. Scale bars: 50 µm; 15 µm (insets in B).

The microstructure of the composite 3D hydrogel construct was assessed using histological staining on longitudinal paraffin sections. The construct was compared to patient-derived parietal peritoneum biopsies from the uterovesical fold to determine how closely it resembled the histological and phenotypical features of the peritoneum. Histological analysis of constructs generated using LP-9/NHDFs or HPMCs/HPFs revealed a cell monolayer residing on the surface of the matrix, which was interspersed with fibroblastic cells. This 3D arrangement mimicked the peritoneal layer; however, the submesothelial layer of the constructs contained fewer cells than that of human parietal peritoneum (Fig. 3A; Fig. S3). This finding might be caused by experimental variation in the cell density within hydrogels due to uneven distribution of the cells during model preparation (Fig. S4). PDPN, MSLN and CK were expressed in the surface layer of the LP-9/NHDFs or HPMCs/HPFs constructs, comparable to the mesothelial layer of the parietal peritoneum, confirming their mesothelial origin (Fig. 3A; Fig. S3A) (Braun et al., 2011). As in the parietal peritoneum, we also detected collagen IV (COLIV; also known as COL4) expression in a thin layer beneath the mesothelium in the constructs, indicating the spontaneous formation of a basal lamina by LP-9 and HPMC (Fig. 3B; Fig. S3B). Cells cultured within the matrix expressed FSP1, TEM1 and αSMA, and were comparable to those in the submesothelial layer of the parietal peritoneum (Fig. 3A; Fig. S3B). In constructs using LP-9 cells, we found that they expressed FSP1, αSMA and TEM1, suggesting that these markers were upregulated in LP-9 cells under these culture conditions. Interestingly, LP-9 cells cultured in 2D under different media compositions led to variation in FSP1 and αSMA expression (Fig. S5), suggesting that the expression profile of LP-9 cells was influenced by the culture environment. Therefore, we used patient-derived HPMCs and HPFs in further analysis of composite 3D hydrogel constructs as simple peritoneal layer models.

To determine the functionality of the composite 3D hydrogel construct as a peritoneal layer model, we assessed the level of human tissue plasminogen activator (tPA; also known as PLAT) (van Hinsbergh et al., 1990; Whitaker et al., 1982) in conditioned media from the apical compartments of transwell inserts carrying HPMC/HPF constructs (Fig. 3C). Human tPA was detected from day 1 over the 10-day culture period using constructs from three individual HPMCs donors, with an average peak on day 5 (11.09±5.43 ng/ml), in line with reported tPA levels in cultured mesothelial cells (van Hinsbergh et al., 1990). The tPA level was lower in donor 1 construct after 10 days of culture. This might be caused by higher cell damage as detected by higher LDH release in donor 1 construct (Fig. 2D and Fig. 3C). Overall, these results suggest that the HPMCs retain classical mesothelial characteristics in the peritoneal layer model.

### Endometrial assembloids contain the two main endometrial cellular subtypes, representing the basic endometrial cellular composition

**Fig. 4.**
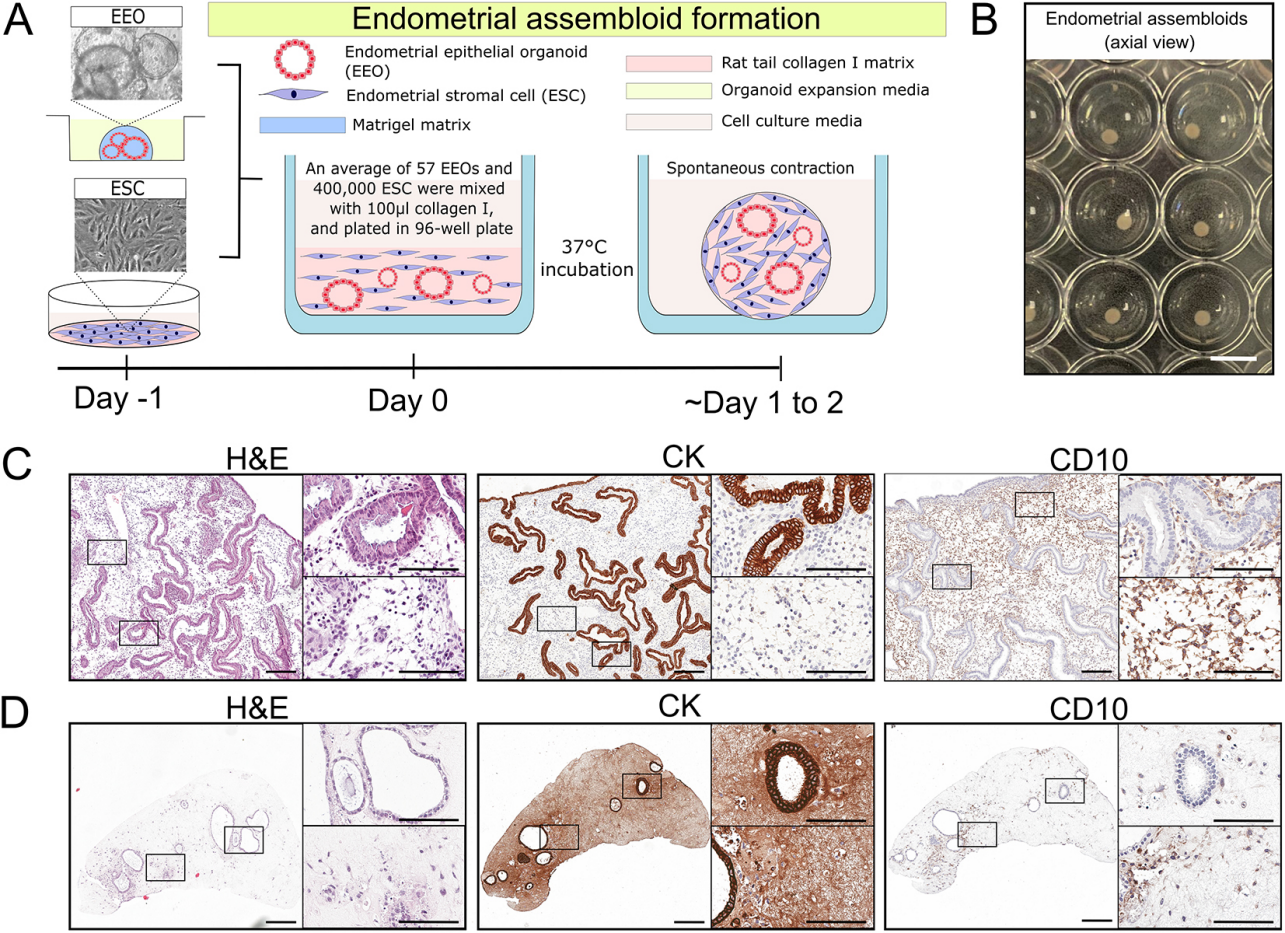
**Assembly and characterisation of endometrial assembloids from endometrial epithelial organoids and endometrial stroma.** (A) Schematic illustration of endometrial assembloid formation. (B) Representative axial view of contracted endometrial assembloids in a 96-well plate. (C,D) Histological staining of the cytoskeletal markers cytokeratin (CK) and CD10 on transverse sections of the eutopic endometrium (C) and endometrial assembloids (D). Scale bars: 3.2 mm (B); 200 µm (C,D); 100 µm (insets in C,D).

To constitute the endometrial component of superficial endometriotic lesions, endometrial assembloids were generated from patient-derived cells. Endometrial epithelial organoids (EEOs) and endometrial stromal cells (ESCs) were combined in a collagen I hydrogel. We observed that both cell types within the hydrogel spontaneously contracted to form spherical assembloids with an average diameter of 1.15±0.07 mm (Fig. 4A,B). Histological analysis confirmed that our endometrial assembloids retained both glandular and stromal components in a 3D configuration in line with previous studies (Liao et al., 2024; Rawlings et al., 2021). These constructs preserved endometrial glandular elements surrounded by endometrial stroma (Fig. 4C), with the purpose to serve as mimic of shed menstrual endometrial fragments (Shih et al., 2022). EEOs expressed CK and retained a luminal space within the assembloids, while ESCs expressed CD10 and were distributed throughout the assembloids (Fig. 4D).

### Endometrial assembloids transplanted onto the peritoneal layer model mimic early superficial endometriosis formation

**Fig. 5.**
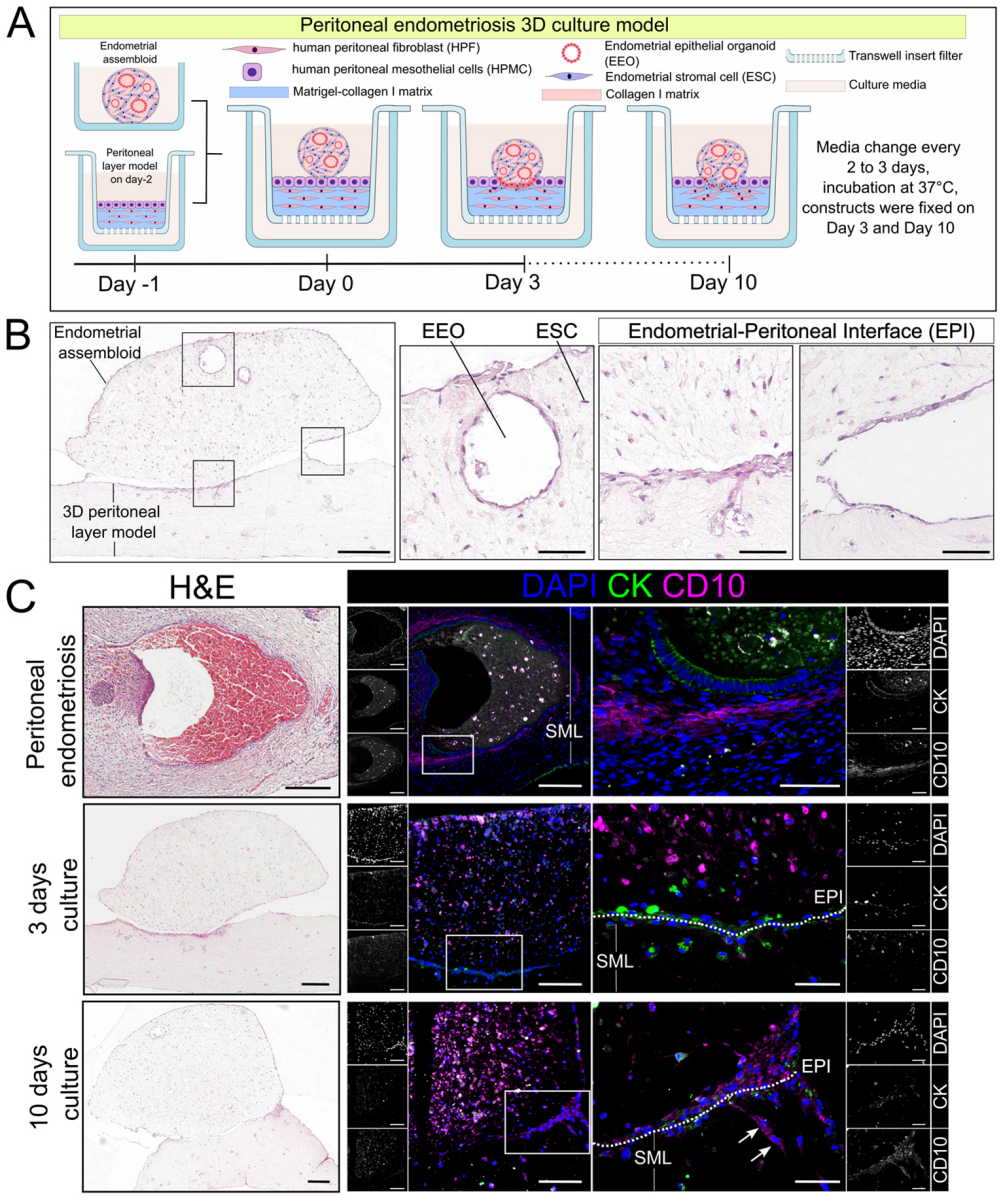
**Characterisation of the superficial endometriosis model.** (A) Schematic illustration of superficial endometriosis model assembly, comprising an endometrial assembloid and peritoneal layer model. (B) H&E staining on transverse section of a superficial endometriosis model highlighting endometrial and peritoneal components, as well as the EEOs, ESCs and endometrial-peritoneal interface (EPI). (C) Histological staining and immunofluorescence of superficial endometriosis lesions and *in vitro* model after 3 and 10 days of culture revealed CK^+^ and CD10^+^ cells in the EPI (dotted line) and submesothelial layer (SML). Scale bars: 300 µm (B); 200 µm (C); 50 µm (insets in B,C).

To create an *in vitro* model of superficial endometriosis, endometrial assembloids were transplanted onto peritoneal layer models. The combined models were analysed on days 3 and 10 of co-culture (Fig. 5A). Overall model architecture revealed that endometrial assembloids adhered to the apical surface of the peritoneal layer model, and a continuous layer of cells formed covering the assembloid and peritoneal layer model (Fig. 5B; Fig. S6). Furthermore, we observed that EEOs within endometrial assembloids displayed gland-like structures and an endometrial-peritoneal interface (EPI), where endometrial assembloids and the peritoneal layer attached (Fig. 5B). We compared the endometriosis models with a peritoneal endometriosis lesion that showed the presence of epithelial cells with CK^+^ gland-like structures surrounded by CD10^+^ cells within the submesothelial layer of the peritoneum (Fig. 5C). In the 3D peritoneal endometriosis model, the EPI was composed of cells expressing CK and CD10, suggesting the presence of epithelial cells (HPMCs or EEOs) and ESCs (Fig. 5C). However, CK expression appeared less well organised and was reduced by day 10, suggesting disruption of the mesothelial cell boundary. At this stage, CD10^+^ ESCs were more abundant at the EPI, with some CD10 cells observed within the peritoneal wall beneath the EPI (Fig. 5C), indicating that a subset of these cells had begun to infiltrate the peritoneal layer of the model.

These findings indicate that the endometrial assembloid and the peritoneal wall components interacted to form a defined EPI, at which ESC appeared to migrate into the peritoneal compartment. Furthermore, a continuous epithelial layer across the entire construct was present. These observations suggest that the model recapitulated early cellular events associated with endometriotic lesion establishment *in vitro*.

## DISCUSSION

**Fig. 6.**
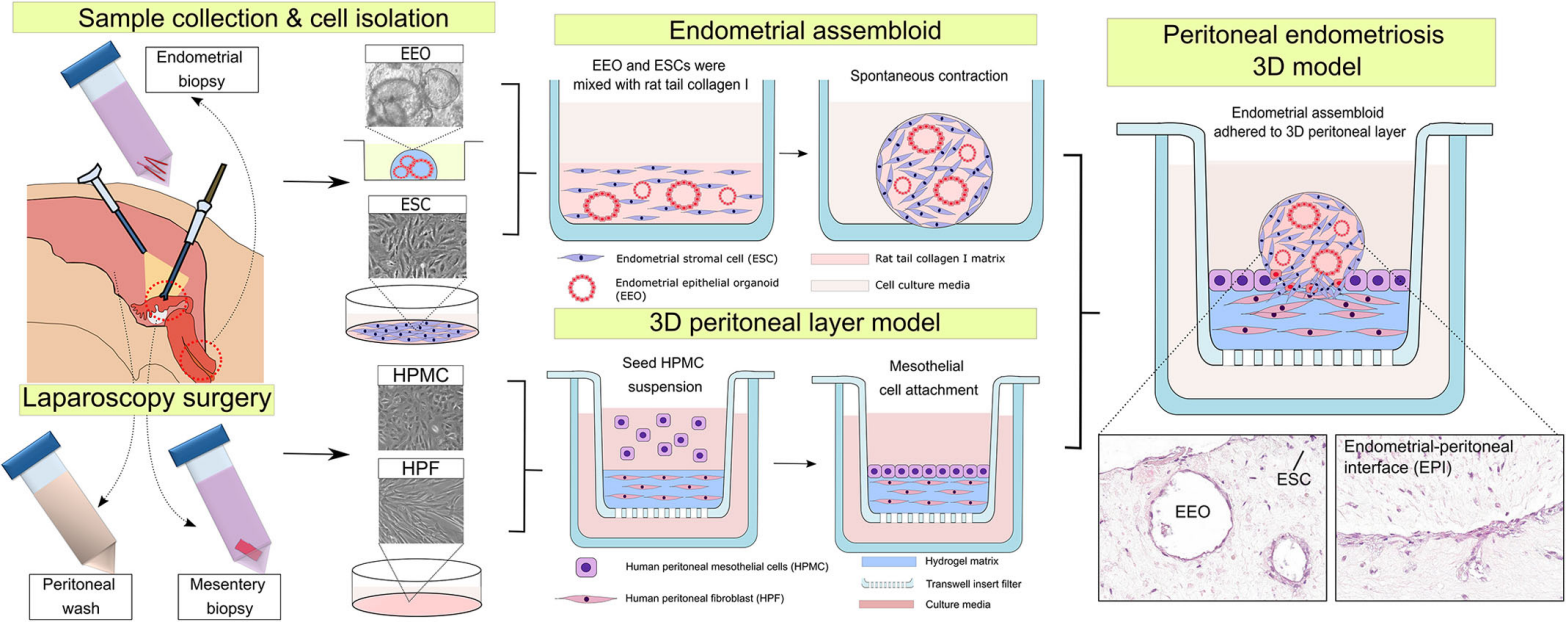
**Schematic overview of the workflow used to establish the *in vitro* peritoneal endometriosis model.** Routine laparoscopy provided peritoneal wash fluids, peritoneum from the fallopian tube mesentery and endometrial biopsies, which together served as the sources for constructing a multicomponent model of peritoneal endometriosis.

In this study, we have demonstrated that patient-derived primary cells can be utilised to establish a 3D cell culture system with which to model superficial peritoneal endometriosis formation. We observed that different cell types emerged in close vicinity to each other during the process of establishing the multicellular complex model. Specifically, we demonstrated that endometrial assembloids attached to and interacted with the peritoneal layer model. Importantly, our cell isolation technique is pragmatic as all needed biosamples are easily harvested from a single patient undergoing routine laparoscopic surgery without the need for any additional or high-risk invasive procedures that compromise patient safety. Peritoneal wash fluid, excised peritoneum and endometrial biopsies are common byproducts of routine laparoscopy surgeries, and provided the materials required to generate a multicomponent model of peritoneal endometriosis (Fig. 6). Therefore, this study supports future development towards more ethically sound clinical applications (Emanuel et al., 2000) and personalised disease modelling.

The peritoneum plays an important role as the first contact point for endometrial tissue fragments in endometriosis lesion initiation based on the retrograde menstruation theory (Hapangama et al., 2012; Sourial et al., 2014). Therefore, the peritoneal model is of great value in further exploring the cellular and molecular mechanisms involved in early endometriosis formation, which can be utilised to develop preventative strategies in the future.

With regards to the advancement of 3D cell culture techniques, a recent study has generated an *in vitro* endometriosis model by combining spheres of endometrial epithelial and stromal cell lines with an LP-9 mesothelial monolayer (Song et al., 2023). Although peritoneal endometriosis models using established cell lines are easier to construct, they fail to fully replicate the cellular heterogeneity and phenotypic complexity observed *in vivo* (Marr et al., 2025). In this study, we generated a co-culture system with a peritoneal element composed of both mesothelial and submesothelial layers from patient-derived cells. Moreover, our endometrial model comprised EEOs surrounded by ESCs, a configuration that resembles the cellular organisation observed in shed endometrial fragments in menstrual effluent (Shih et al., 2022). Our novel model recapitulates both peritoneal and endometrial architecture and is replicable and expandable.

The challenge in developing a patient-derived *in vitro* peritoneal layer model was to maintain a flat-shaped structure to be readily implanted with multicellular mimics of endometrial fragments. Our model was designed to address these limitations and enabled sustained endometrial-peritoneal interactions for a 10-day period, exceeding the duration achievable in existing systems (Song et al., 2023). Collagen I is the most abundant extracellular matrix protein in the submesothelial layer (Kastelein et al., 2019; Witz et al., 2001). Therefore, the utilisation of collagen I-based hydrogels as extracellular matrix mimetics could mimic the native cellular environment and support fibroblast viability *in vitro*. However, fibroblast-collagen interactions often resulted in hydrogel contraction, which disrupts the mesothelial layer (Zhang et al., 2019). Previous studies suggested that contraction of the fibroblast-collagen mixture could be minimised vertically by using alginate cross-linkage and horizontally by polydopamine adhesion (Kim et al., 2022). Our study found that a collagen I-Matrigel matrix minimised gel contraction in the peritoneal layer model. The use of Matrigel increases hydrogel stiffness owing to the formation of larger pores that increase rigidity (Anguiano et al., 2020). The contribution of Matrigel to overall hydrogel composition was limited to a 50:50 (v/v) ratio, as Matrigel is more commonly used to model cancer pathogenesis. Therefore, increasing the Matrigel ratio may minimise the resemblance of our 3D peritoneal layer model to imitate the healthy peritoneal extracellular matrix. Further analysis is required to determine the rheological properties of the peritoneal layer model in comparison with those of healthy peritoneal tissue.

The methods we have developed in biosample procurement and 3D culture systems allow further application beyond superficial endometriosis modelling; other endometriosis subtypes, such as deep endometriosis and ovarian endometriosis, may be modelled in the future. This may include the application of pro-inflammatory cytokines and an extended culture period beyond 10 days to facilitate such modelling. As the peritoneum is instrumental in the development of other pathologies, such as cancer metastasis, the peritoneal layer model can be utilised for diverse disease modelling pursuits. Ovarian cancer metastasis has been previously modelled by incorporating patient-derived omental adipocytes and various cell lines such as MeT5A mesothelial cells, MRC-5 fibroblasts, EA.hy926 endothelial cells and THP-1 macrophages (Estermann et al., 2023). We anticipate that our patient-derived model can be expanded with the addition of immune cells and endothelial cells to study conditions such as metastasis. Further work that will benefit from the prototype we present here, include studies exploring trans-mesothelial water permeability in peritoneal dialysis (Piccapane et al., 2021) and those examining inflammation (Zhang et al., 2022). Besides, our model secreted tPA, which activates plasminogen in the serosal cavity and plays important roles in peritoneal adhesion formation (Mutsaers et al., 2016). This function has been previously modelled to explore wound healing and fibrotic processes using MeT5A mesothelial cells, but lacked tPA assessment (Ditzig et al., 2022), suggesting the utility of our model to improve this study in the future. Therefore, our peritoneal layer model is a promising platform to be modified into a more complex mimic of *in vivo* lesions, using patient-derived primary cells, and may be applicable to diseases other than endometriosis.

Our superficial endometriosis 3D model enabled the identification of an EPI, where endometrial assembloids interacted with the peritoneal layer. This interface represents a dynamic zone of cellular exchange, characterised by the apparent migration of ESCs into the peritoneal compartment, and formation of an epithelial covering of the entire construct. These findings provide a valuable platform for future studies investigating the cellular and molecular mechanisms underlying early endometriotic lesion establishment, including the potential contributions of different cell types and their involvement in epithelial-mesenchymal transition and mesothelial-mesenchymal transition processes. As tissue analysis technologies continue to rapidly develop, the molecular mechanisms involved at the EPI may be further studied through spatial transcriptomics, as has previously been performed in actual endometriosis lesions and the eutopic endometrium (Liu et al., 2025; Tempest et al., 2025). Moreover, our study demonstrated the utility of conditioned media to assess cell viability and mesothelial functioning in the peritoneal layer model. Therefore, this platform offers the possibility of assessing cytokines, chemokines and growth factors involved in early endometriosis formation using the conditioned media, as previously assessed in endometriosis patient samples (Han et al., 2023). However, such analyses would be improved by incorporating immune cells into the superficial endometriosis model, because endometriosis is a complex disease involving the peritoneal immune microenvironment (Chen et al., 2023).

Regarding limitations, this study was conducted using peritoneal fibroblasts with some degree of mesothelial cell contamination, which may interfere with the cellular composition of the submesothelial layer. The purity of the peritoneal fibroblast isolates could be improved by cell sorting, as further refinement using more purified cell populations could enhance the specificity and mechanistic insights of the model. Future studies may include bulk or single-cell RNA sequencing to confirm the identity of the primary peritoneal mesothelial and fibroblast cells, and 3D imaging to provide comprehensive insight into the entire model structure. A further limitation is the currently minimal functional assessment of the early endometriosis model. In future studies, it will be important to assess the impact of ovarian sex hormone exposure on the model, as previously described (Song et al., 2023). Deep characterisation of the endometriosis model in comparison to patient-derived early endometriosis lesions using omics technologies will allow assessment of how far the model recapitulates *in vivo* lesions in composition and physiological functioning.

### Conclusions

Peritoneal superficial endometriosis causes debilitating chronic pelvic pain and other symptoms that negatively affect the wellbeing and productivity of millions of women of reproductive age worldwide. There is no curative treatment, and diagnosis requires invasive surgical interventions. Understanding endometriosis pathophysiology and identifying diagnostic and therapeutic targets are hampered by the challenges in examining disease initiation and progression. Therefore, a physiomimetic patient-derived *in vitro* model that accurately mimics the lesions of peritoneal endometriosis is urgently needed. Such a model will overcome the bottleneck that currently exists in the endometriosis discovery pathway. Herein, we present an *in vitro* model not only containing the main cell types of both the peritoneal and endometrial components of endometriotic lesions but also demonstrating interactions between the cell components, thus providing a robust platform for further refinement for the benefit of endometriosis research.

## MATERIALS AND METHODS

### Ethical approval

The collection and use of human tissue was approved by the Liverpool Adults Ethics Committee (REC:19/SC/0449; approval date 7 September 2019). All women involved provided written informed consent to the use of their tissue biopsies and relevant clinical information. This study was conducted in accordance with the ethical principles of the Declaration of Helsinki.

### LP-9 and NHDF cell culture

The human mesothelial cell strain LP-9 (AG07086) was originally isolated from the peritoneal cavity of a patient with ovarian cancer and was purchased from the Coriell Institute (Camden, NJ, USA). LP-9 cells were cultured in mesothelial culture medium consisting of 1:1 (v/v) Medium 199 (Sigma-Aldrich, M4530)/MCDB105 (Sigma-Aldrich, 117-500). The medium was supplemented with 2 mM L-glutamine (Sigma-Aldrich, G7513), 15% (v/v) foetal bovine serum (FBS; Merck, F7524), 10 ng/ml human epidermal growth factor (Lonza, CC-4107), 0.4 µg/ml hydrocortisone (Sigma-Aldrich, H0888) and 100 µg/ml Primocin (Invivogen, ant-pm-2).

NHDFs (C-12300) isolated from the dermis of juvenile foreskin were purchased from Promocell (Heidelberg, Germany). NHDFs were cultured in Fibroblast Growth Medium (Promocell, C-23110) containing 1 ng/ml recombinant human basic fibroblast growth factor and 5 μg/ml recombinant human insulin, supplemented with 2 mM L-glutamine and 100 µg/ml Primocin. All cells were cultured on 0.1% (w/v) gelatine-coated plates at 37°C with 5% CO_2_.

### Biosample collection

Peritoneal wash fluid (PWF), fallopian tube mesentery, eutopic endometrium and endometriosis lesion biopsies were collected from women undergoing laparoscopic surgery for benign conditions at Liverpool Women's Hospital. Information on participants' demographics, samples collected and the experiments in which their samples were utilised is presented in Table S1.

### Primary peritoneal mesothelial cell isolation and culture

HPMCs were collected from PWF following a previously described method (Holl et al., 2021) with some modifications. PWF was collected under sterile conditions during peritoneal cavity wash with saline solution and centrifuged at 300 ***g*** for 10 min to obtain a cell pellet. Ficoll-Paque (Sigma-Aldrich, GE17-1440-02) was used to remove red blood cells from the sample. The HPMC cell pellets were frozen in Recovery Cell Culture Freezing Medium (Gibco, 12648010) and stored at −150°C before use. HPMCs were cultured under the same conditions as LP-9 cells, with a seeding density of 1.67×10^5^ cells/cm^2^ (Fig. S7).

### Primary peritoneal fibroblast cell isolation and culture

HPFs were isolated from fallopian tube mesentery biopsies following a previously described method (Witowski and Jörres, 2006) with some modifications. Briefly, fallopian tube mesenteries were initially washed with sterile PBS (Gibco, 10010-015) three times, then incubated in 1× Trypsin-EDTA (Sigma-Aldrich, T4174) in PBS for 20-30 min at 37°C in a shaking water bath to remove the mesothelial layer. This step was repeated twice before cutting the mesentery biopsies into small explants (1-2 mm) using scalpel blades. The explants were plated in a six-well plate and covered with 1.5-2 ml Fibroblast Growth Medium. When cells had started growing out of the explants and reached 20-30% confluency, the explant pieces were removed and discarded to minimise further mesothelial contamination. Medium was changed every 2-3 days. Upon reaching 90-95% confluency, HPFs were detached from the plate using 1× Trypsin-EDTA, frozen in Recovery Cell Culture Freezing Medium and stored at −150°C. For further expansion, HPFs were cultured under the same conditions as NHDFs, with a seeding density of 1.67×10^5^ cells/cm^2^ (Fig. S7), except that they were cultured on non-coated plates to minimise mesothelial cell adherence.

### Isolation of primary endometrial epithelium and stroma

Human endometrial epithelium (glands) and stroma were isolated from endometrial pipelle biopsies as previously described (Valentijn et al., 2013). Endometrial tissue was cut into small fragments (<1 mm) using a scalpel blade and further enzymatically digested with 1 mg/ml Dispase II (Gibco, 17105041), 2 mg/ml collagenase type I (Gibco, 17100017) and 80 µg/ml deoxyribonuclease (DNase) I (Merck, 11284932001) for 40 min to 1 h at 37°C in a shaking water bath. The digests were triturated every ∼15 min to facilitate tissue breakdown into free epithelial glands and single stromal cells. The endometrial digests were then filtered through a 40 µm cell strainer (Corning, CLS431750) to separate the glandular (retentate) and stromal (flow-through) components. Erythrocytes were removed from the endometrial stromal fraction using Ficoll-Paque. The intact endometrial glands and stromal cells were frozen in Recovery Cell Culture Freezing Medium and stored at −150°C until use.

### EEO expansion

EEOs were derived from the isolated endometrial glands following the method by Turco et al. (2017). Frozen isolated endometrial gland fragments were thawed at 37°C and washed in 4 ml Dulbecco's modified Eagle medium (DMEM)/Ham's F-12 (Gibco, 21041033). Subsequently, endometrial gland fragments were mixed with ice-cold Matrigel (Corning, 536231) at 1:20 (v/v) ratio and plated as 20 µl droplets in 48-well plates. Matrigel droplets were allowed to solidify at 37°C for 15 min, followed by the addition of 250 μl organoid expansion medium (Table S3) per well. The expansion medium was refreshed every 2-3 days, and EEOs were passaged when dense. At passage 2-3, EEOs were removed from Matrigel using Cell Recovery Solution (Corning, 354253) for endometrial assembloid generation.

### ESC culture

ESCs were cultured in DMEM/Ham's F-12 medium supplemented with 10% FBS and 100 µg/ml Primocin. ESCs were cultured on non-coated plates at 37°C with 5% CO_2_, with a minimum seeding density of 3.5×10^4^ cells/cm^2^.

### Endometrial assembloid construction

Endometrial assembloids were generated by embedding EEOs and ESCs at p2-3 into a collagen I hydrogel (Gibco, A-10483), which was prepared according to the manufacturer's protocol. To make a single endometrial assembloid, EEOs were isolated from a single 20 µl Matrigel droplet (average of 57 EEOs), and 4×10^5^ ESCs were mixed with 100 µl collagen I at 2 mg/ml. The mixture was prepared on ice, and 100 µl was deposited per well in a 96-well plate. Subsequently, the EEO-ESC-collagen mixture was set to gelate for 40-60 min at 37°C. Endometrial assembloids were maintained in 200 µl ESC culture medium for 24-48 h, at which point the assembloids had contracted and were harvested for use in the endometriosis model. This method supported stromal cells to remain in the collagen construct. Only a few cells attached to the well following 12 days of culture in a 96-well plate (Fig. S8).

### Peritoneal layer model construction

A 3D peritoneal layer model was generated by stepwise combination of peritoneal mesothelial cells (LP-9 or HPMCs) and fibroblasts (NHDFs or HPFs). Four different hydrogel mixtures (M1-M4) were used in this study (Table 1): M1 (2 mg/ml rat tail collagen I), M2 [2 mg/ml rat tail collagen I and Matrigel matrix at 70:30 (v/v) ratio], M3 [2 mg/ml rat tail collagen I and Matrigel matrix at 50:50 (v/v) ratio] and M4 [2 mg/ml rat tail collagen I supplemented with 10 µg/ml human plasma fibronectin (Merck, FC010)]. Initially, fibroblasts were mixed with rat tail collagen I-based hydrogels at 2000 cells/µl, and 100 µl of the fibroblast-hydrogel mixture was placed in the upper compartment of a 6.5 mm diameter, 3.0 µm polycarbonate membrane transwell insert (Costar, 3415). Subsequently, the transwell insert was placed in a 24-well plate and set to gelate at 37°C for 40-60 min. Next, 100 µl of a cell suspension containing LP-9 or HPMCs in mesothelial culture medium (250 cells/µl) was seeded onto the upper compartment (apical) of the transwell insert, covering the fibroblast-hydrogel mixture. Fibroblast Growth Medium was added to the lower compartment (basolateral) of the well to maintain NHDF/HPF nutrients and prevent dehydration. The 3D peritoneal layer model was cultured at 37°C with 5% CO_2_. Medium was refreshed every 2-3 days, and the conditioned medium was collected at days 1, 3, 5, 7 and 10 for analysis.

**Table 1.** Rat tail collagen I-based hydrogel mixture ratio for 3D peritoneal model construction

Hydrogel mixture	Collagen I*	Matrigel^‡^	Fibronectin^§^
M1	100	0	0
M2	70	30	0
M3	50	50	0
M4	99	0	1

*2 mg/ml rat tail Collagen I (Gibco, A-10483). ^‡^Matrigel matrix basement membrane (Corning, 536231). ^§^1 mg/ml human plasma fibronectin (Merck, FC010).

### Peritoneal endometriosis model construction

A peritoneal endometriosis model was generated by transplanting an endometrial assembloid onto the peritoneal layer model. Initially, the culture media on the apical and basolateral compartments of the peritoneal layer cultures were discarded. The endometrial assembloid was gently extracted from the 96-well plate using manual pipetting and placed onto the surface of the peritoneal layer model. The superficial endometriosis model was maintained under peritoneal layer model culture conditions.

### Tissue biopsy and *in vitro* model processing for histology

Human tissue biopsies and 3D cell culture constructs were fixed in 10% neutral buffered formalin (Sigma-Aldrich, HT501128) overnight (tissue biopsies) or for 30 min (*in vitro* models) at room temperature (RT). The peritoneal layer and peritoneal endometriosis model constructs were detached from the transwell insert by cutting out the filter membrane using a scalpel blade. Constructs were set in Histogel (Fisher Scientific, 12006679) to maintain their shape during the embedding process. All tissue biopsies and the 3D models were processed to paraffin, sectioned (3 μm thickness) and placed on 3-aminopropyltriethoxysilane-coated glass slides for histological and immunostaining analysis.

### Haematoxylin and Eosin (H&E) staining

Sections were deparaffinised in xylene and rehydrated using an ethanol gradient. Sections were stained with Epredia™ Shandon Gill 2 Haematoxylin (Fisher Scientific, 10096648) and Epredia™ Shandon Eosin-Y (Fisher Scientific, 10188418), dehydrated in ethanol and xylene, and mounted in Epredia™ Consul-Mount™ medium (Fisher Scientific, 9990441).

### Immunohistochemistry (IHC)

IHC was conducted as described previously (Kamal et al., 2016). In short, deparaffinised sections underwent antigen retrieval and were incubated with primary antibodies (Table S4) overnight at 4°C. Sections were then washed thoroughly in Tris-buffered saline (TBS) before applying horse radish peroxidase-conjugated ImmPRESS anti-mouse IgG (Vector Laboratories, MP-7402) or anti-rabbit IgG (Vector Laboratories, MP-7401) secondary antibodies for 30 min at RT. Antibody detection was visualised by incubating the sections for 10 min at RT with 3, 3′-diaminobenzidine (DAB; ImmPACT Vector Laboratories, SK-4105). Samples were counterstained using Haematoxylin, dehydrated and mounted as described above.

### Immunofluorescence (IF)

LP-9, NHDFs, HPMCs and HPFs were cultured in eight-well Nunc™ Lab-Tek™ II Chamber Slides™ (Thermo Fisher Scientific, 154534) at a seeding density of 15,000-20,000 cells/well, at 37°C until 80-90% confluency. Cells were fixed in −20°C absolute ethanol for 10 min followed by two gentle PBS washes. Cells were permeabilised with 0.25% (v/v) Triton X-100 (Thermo Fisher Scientific, 85111) in PBS for 10 min at RT and blocked with 2% (w/v) bovine serum albumin (BSA; Sigma-Aldrich, A3803) for 1 h at RT. Cells were incubated with primary antibodies overnight at 4°C (Table S4), followed by three 15 min PBS washes and incubation with secondary antibodies AlexaFluor™ 488 goat-anti-mouse IgG1 (Invitrogen, A21121) and AlexaFluor™ 568 goat-anti-rabbit (Invitrogen, A11011) at 1:1000 dilution, and DAPI (Sigma-Aldrich, D9542) in a 1:500 dilution, for 1 h at RT. The slides were mounted using Fluoromount™ (Invitrogen, 00-4958-02). For IF of the 3D peritoneal layer model, sections were prepared and treated with primary antibodies (Table S4) and AlexaFluor™-labelled secondary antibodies with DAPI at the concentrations mentioned above. An Autofluorescence Quenching Kit (Vector Laboratories, SP-8400-15) was applied for 5-10 min at RT prior to imaging.

### LDH assay

To assess cell viability, LDH release was quantified in conditioned media of the mixed basolateral and apical compartments of the transwell insert using an LDH cytotoxicity assay kit (Roche, 11644793001). Samples were assayed in duplicate following the manufacturer's protocols. Optical density was measured at 490 nm using a FLUOstar Omega microplate reader (BMG Labtech) and fresh culture medium as control.

### Enzyme-linked immunosorbent assay (ELISA)

Human tPA was measured in the conditioned medium obtained from the apical compartment of the 3D cell culture system using a human tPA DuoSet ELISA kit (Biotechne, DY7449-05), following the manufacturer's protocol. Standard curves (Fig. S9) were generated from twofold serial dilutions of tPA standard solution. Samples were assayed in duplicate by measuring optical density at 450 nm using a FLUOstar Omega microplate reader as previously described (Busch et al., 2024).

### Imaging and data analysis

Stained sections were digitalised using an Aperio CS2 slide scanner (Leica Biosystems, 23CS100CE). The cell shape of growing cultures was recorded using bright-field microscopy (Leica DM IL), while IF cells and tissue sections were visualised using epifluorescence microscopy (Leica DM2500 and Nikon Eclipse 50i). H&E and IHC images, and the histomorphometry analysis were carried out using Aperio ImageScope Version 12.4.6.5003. Quantification of cell marker expression was performed by manually counting positive cells in three to five regions of interest per well using the Fiji Cell Counter plugin and subsequently calculating the mean percentage±s.d. Axial view images of peritoneal models were acquired using a dissection microscope (Leica MZ16 F). The average endometrial assembloid diameter±s.d. was measured using Fiji. The standard curve of tPA ELISA was generated in GraphPad Prism Version 9.0 using a four-parameter logistic regression model. Data were visualised as graphs using OriginPro 2024b.

## Supplementary Material

10.1242/dmm.052436_sup1 Supplementary information
