# Supplementary material for: Compound design of a patient-derived 3D cell culture system modelling early peritoneal endometriosis
Source: Dis Model Mech. 2026 Feb 2;19(6):dmm052436. doi: 10.1242/dmm.052436 (PMC12919959; doi:10.1242/dmm.052436)
Supplement: Supplementary information [file dmm-19-052436-s1.pdf]

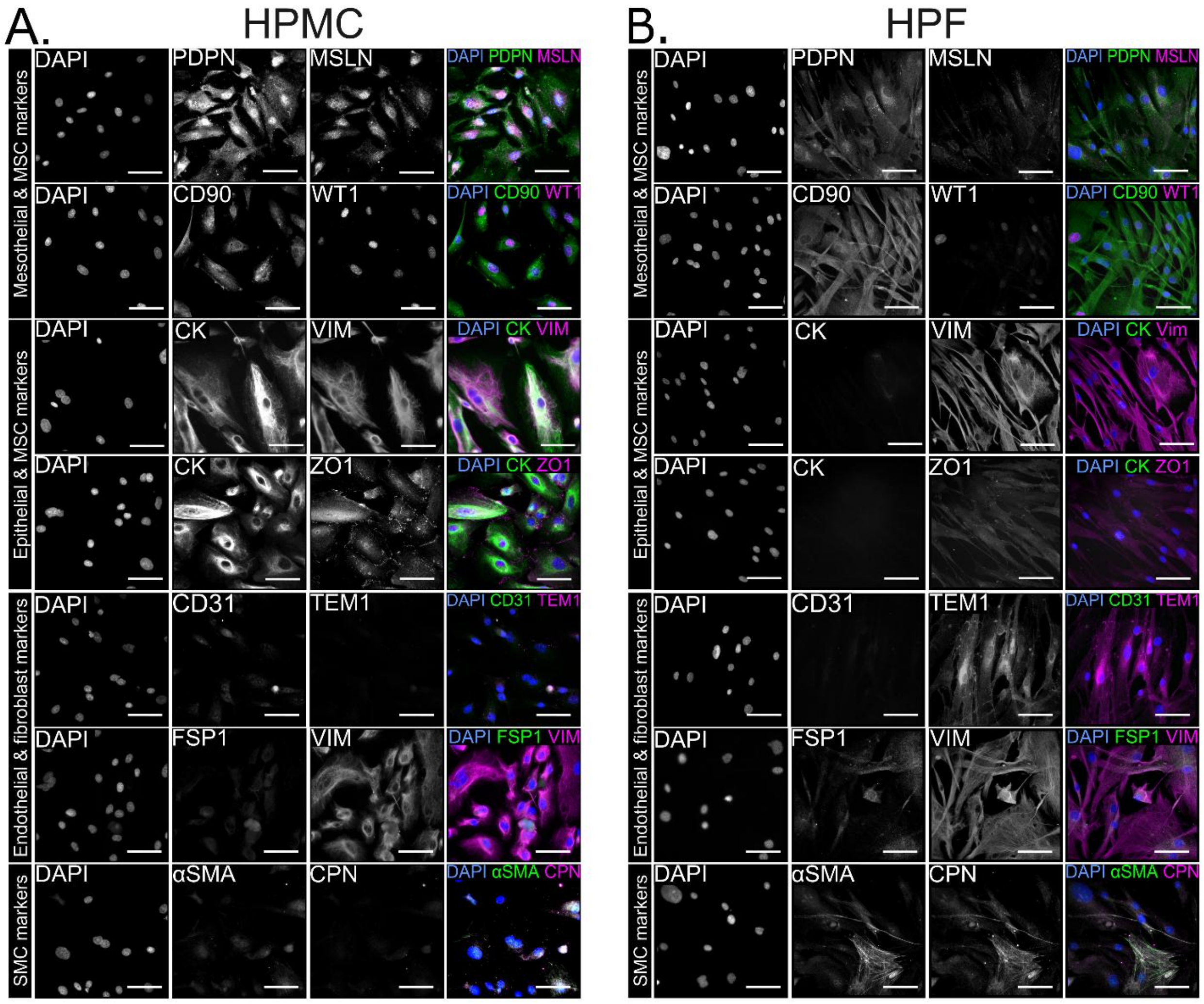

Fig. S1. Characterisation of primary human peritoneal mesothelial cells (HPMCs) and human peritoneal fibroblasts (HPFs) using various markers. (A) HPMCs at passage 2 (p2) express mesothelial cell marker podoplanin (PDPN), mesothelin (MSLN) and WT1; epithelial cell markers cytokeratin (CK) and Zonula Occludens-1 (ZO1), mesenchymal stem cell (MSC) marker CD90, and fibroblast marker Vimentin (VIM). The endothelial cell marker CD31, other fibroblast marker FSP1 and TEM1, and smooth muscle cell (SMC) marker αSMA and calponin (CPN) were found to be absent or at lower expression in HPMCs. (B) HPFs at p2 from the same individual donor express VIM, TEM1, CD90 and αSMA and CPN stress fibres, with the absence of CK, ZO1, and CD31. PDPN, MSLN, and WT1 were observed in HPFs at lower expression compared to HPMCs. Scale bars: 50 μm.

Disease Models & Mechanisms • Supplementary information

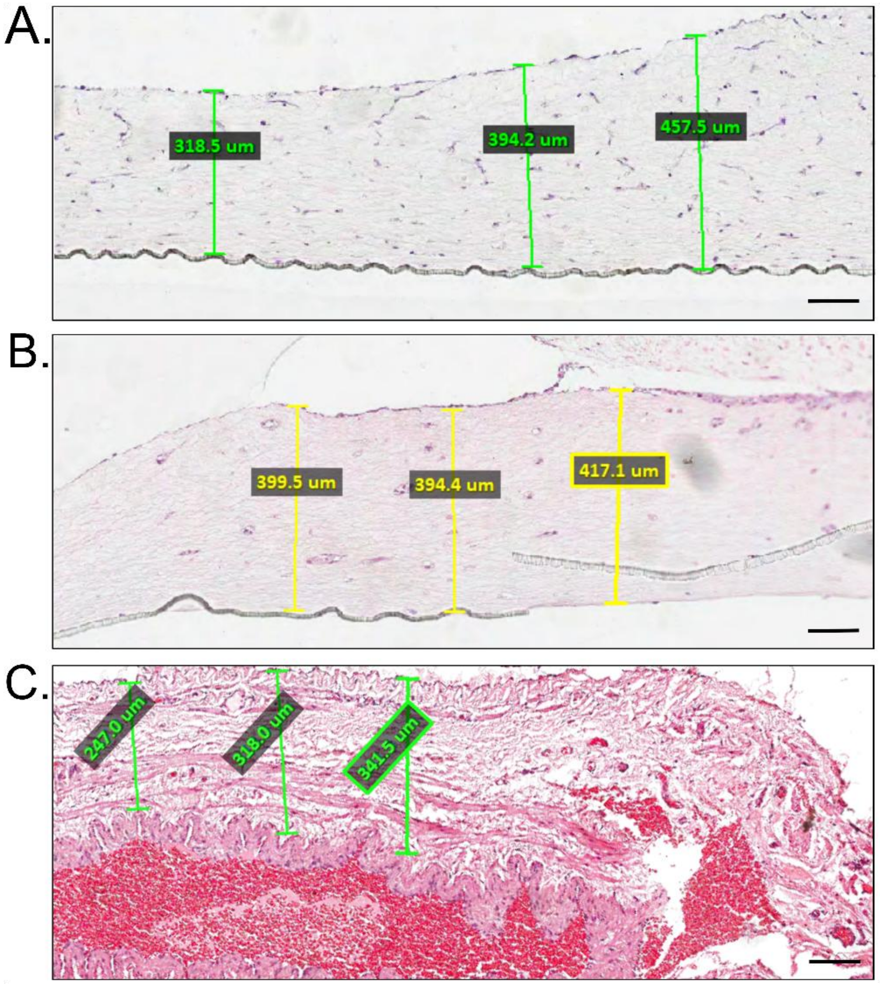

Fig. S2. Representative images showing quantitative histomorphometry of the submesothelial layer thickness. (A) 3D peritoneal layer model in 50:50 collagen I:Matrigel hydrogel matrix; (B) 3D peritoneal endometriosis model; and (C) human parietal peritoneal tissue from the uterovesical fold. Scale bars: 100 µm

Disease Models & Mechanisms • Supplementary information

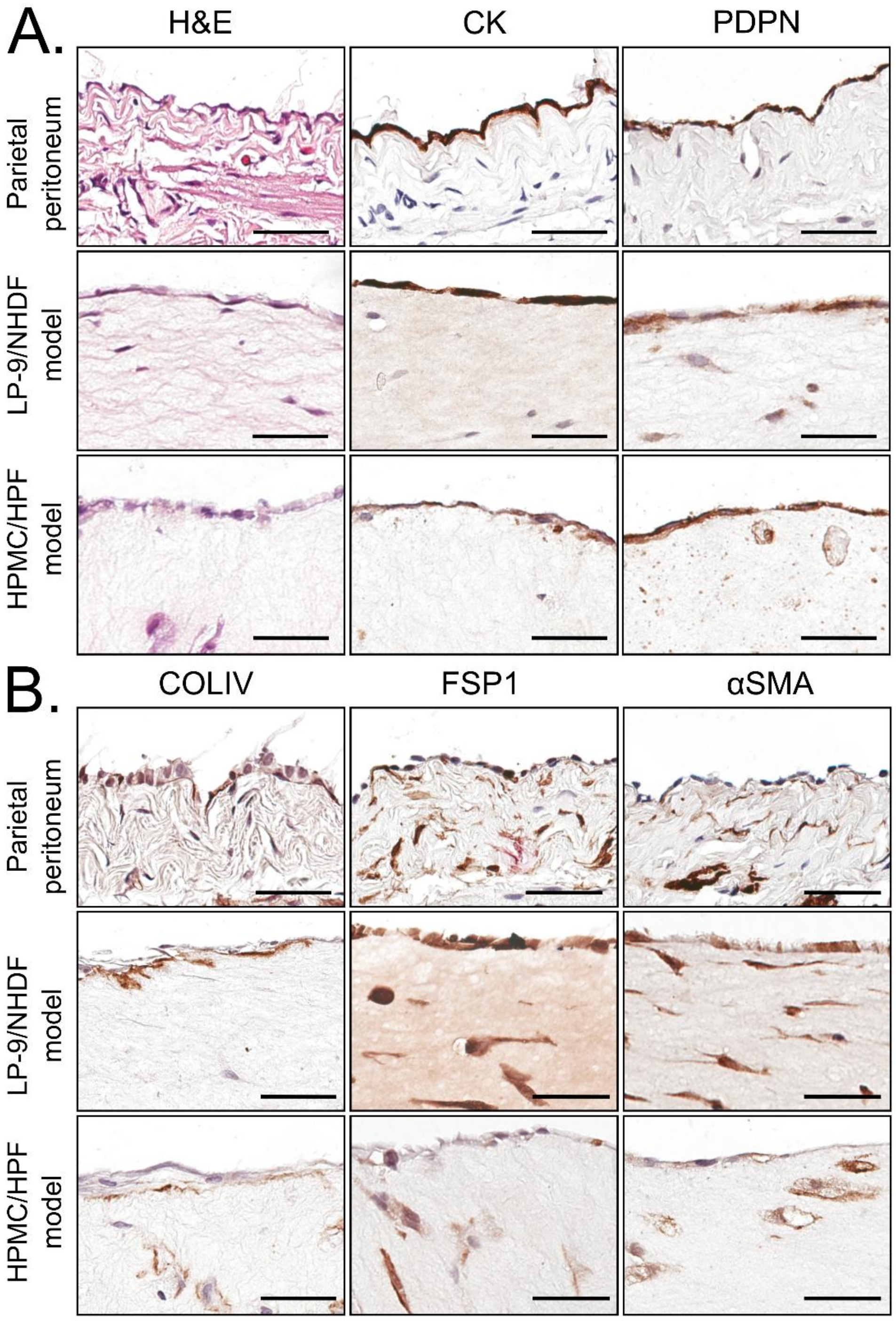

Fig. S3. Histological staining of transverse sections through parietal peritoneum and composite 3D hydrogel constructs composed of LP-9/NHDFs and HPMCs/HPFs. (A) H&E and immunohistochemical staining using antibodies against the cytoskeletal markers cytokeratin (CK) and podoplanin (PDPN); (B) collagen IV (COLIV), fibroblast specific protein 1 (FSP1) and α-smooth muscle actin (αSMA). Scale bars: 100 μm.

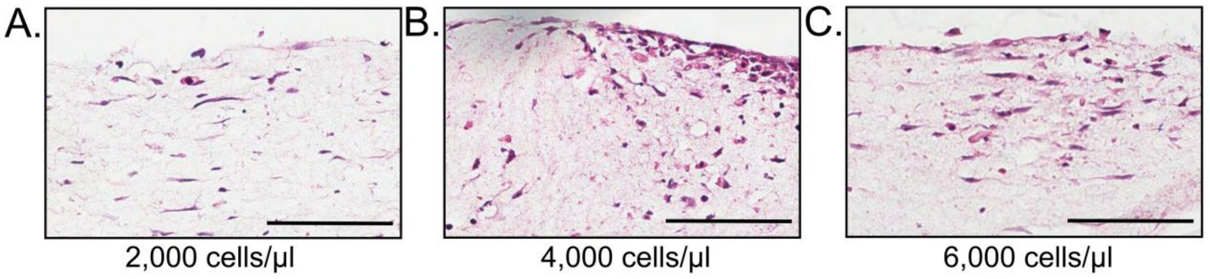

Fig. S4. Variation in cell density across and within constructs. Haematoxylin & eosin staining on transverse section of the fibroblast in 50:50 collagen I:Matrigel hydrogel matrix (combination 3) with various seeding density. Scale bars: 100 µm

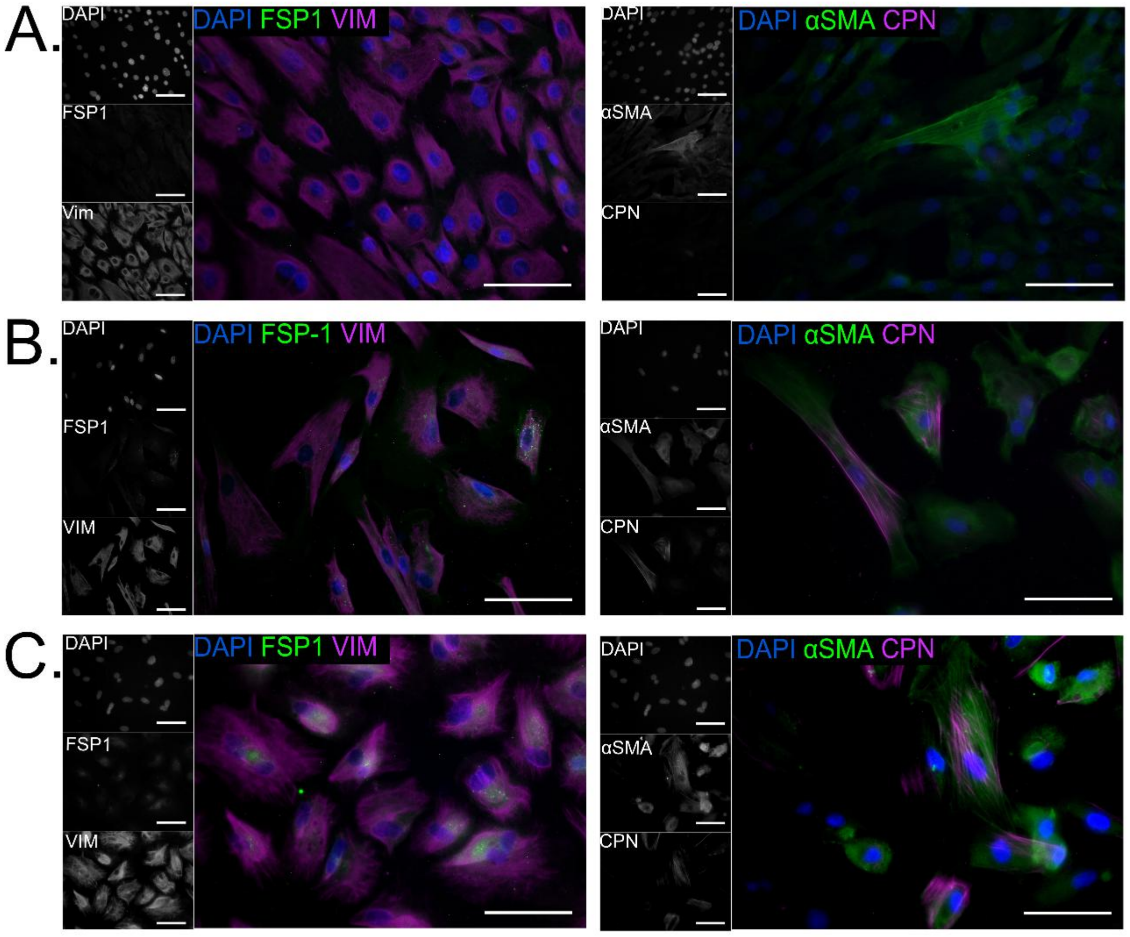

Fig. S5. Variation in marker expression in LP-9 cells under different culture conditions. LP-9 cells at passage 10 (p10) showed variability in fibroblast specific protein-1 (FSP1), alpha smooth muscle actin (αSMA), and calponin (CPN) expression, when cultured under (A) mesothelial culture media (Medium199/MCBD105 (1:1 [v/v]) supplemented with 15% fetal bovine serum (FBS) and 10 ng/ml human epidermal growth factor (EGF)); (B) fibroblast growth medium (Promocell, C-23110) containing 1 ng/ml human basic Fibroblast Growth Factor (bFGF) and 5 µg/ml recombinant human Insulin; (C) Endometrial stromal cell culture medium (DMEM/Ham's F-12 media supplemented with 10% FBS). Scale bars: 50 µm.

Disease Models & Mechanisms • Supplementary information

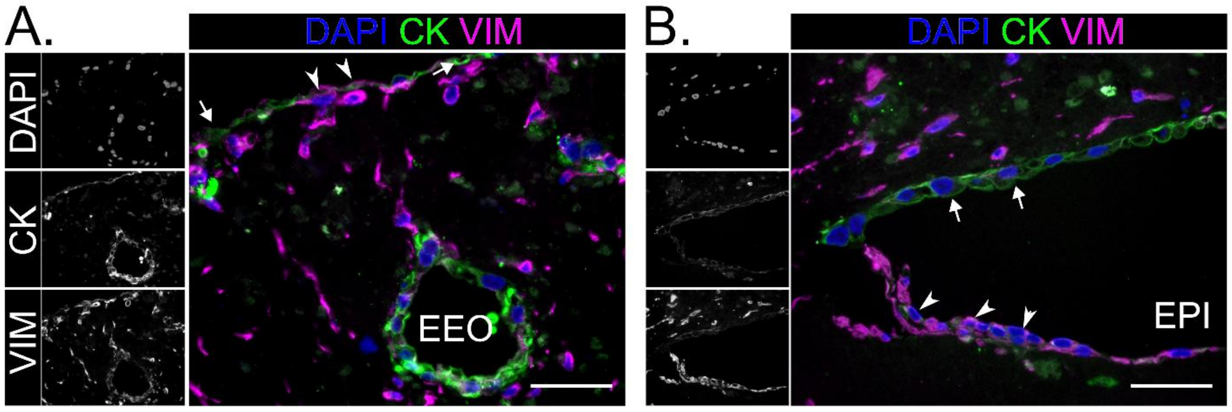

Fig. S6. Immunofluorescence staining of sections through superficial endometriosis model at day 3 using CK and VIM. (A) Epithelial cells expressing CK were detected in EEO and on the surface (arrows) of endometrial assembloids, where some of these CK+ cells co-expressed VIM (arrowheads). (B) Epithelial cells at the edge of the EPI, expressing CK and VIM were found on the surface of the peritoneal layer model, in contact and seemingly continuous with epithelial cells surrounding the assembloid, which lacked VIM expression. Scale bars: 50 μm

Disease Models & Mechanisms • Supplementary information

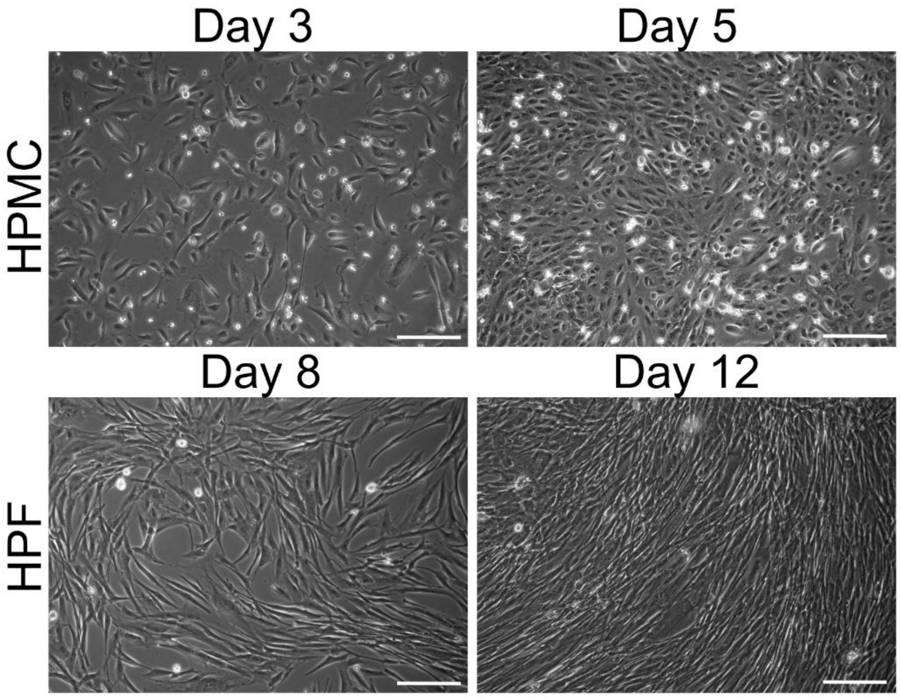

Fig. S7. Human peritoneal mesothelial cell (HPMC) and human peritoneal fibroblast (HPF) cell culture at passage 1 (P1), initial seeding density $1.67x10^5$ cells/cm^2. Scale bars: 200 μm

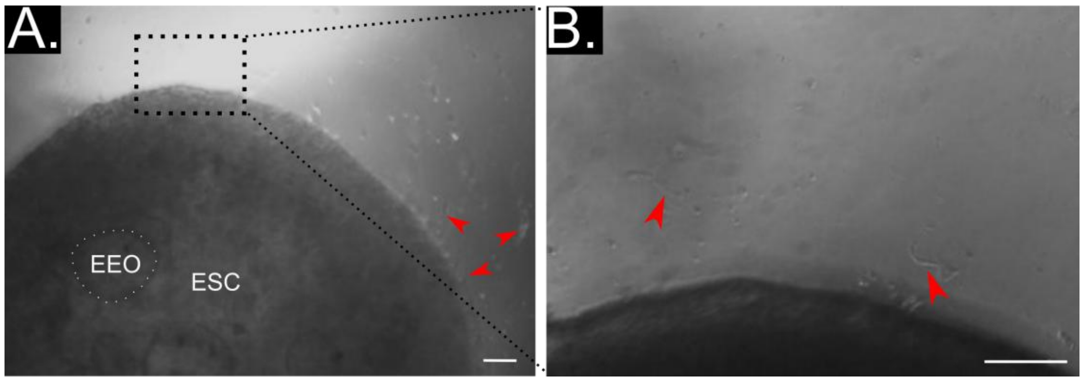

Fig. S8. Microscopic images of the endometrial assembloid at 4x magnification (A) and 10x magnification (B) following 12 days of culture. Endometrial epithelial organoid (EEO) surrounded by the endometrial stromal cells (ESC) in the collagen I hydrogel matrix. Only a few cells (red arrowheads) attached to the well. Scale bars: 200 μm.

Disease Models & Mechanisms • Supplementary information

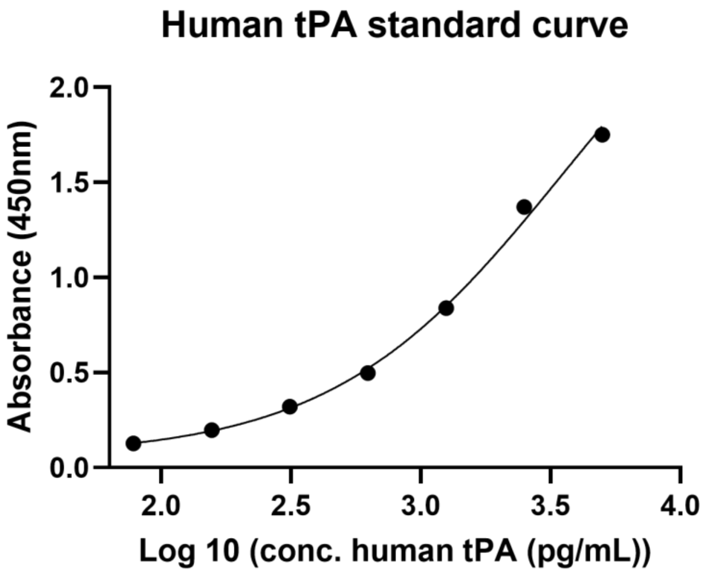

Fig. S9. Standard curve for human tissue plasminogen activator enzyme-linked immunosorbent assay (tPA ELISA).

Disease Models & Mechanisms • Supplementary information

Table S1. Participant demographic

No.	Menstrual phase	Age	BMI	Parity	Reason for surgery	Sample collected					Experiments
						PWF	FTM	EB	UVF	ECT	
1	HT	23	49.9	0	Diagnostic laparoscopy	✓					IF cells, HPMC purity scoring
2	Secretory	28	33.2	0	Diagnostic laparoscopy	✓					IF cells, HPMC purity scoring
3	Proliferative	34	25.2	2	Laparoscopic sterilisation	✓					IF cells, HPMC purity scoring
4	HT	28	27.3	1	Diagnostic laparoscopy +/- excision endometriosis	✓					IF cells, HPMC purity scoring
5	Mid-phase	21	24	1	Ovarian cystectomy	✓					IF cells, HPMC purity scoring
6	Secretory	42	33.1	5	Diagnostic laparoscopy	✓			✓		IF cells, HPMC purity scoring, IF tissue, H&E, IHC, submesothelial measurement
7	Proliferative	37	29	3	Other	✓	✓				IF cells, HPF purity scoring,<br>HPMC and HPF characterisation,<br>3D peritoneal model - donor 3 LDH assay and ELISA
8	Menopause	55	31.5	2	Other		✓				IF cells, HPF purity scoring
9	Secretory	38	20.5	N/A	N/A	✓	✓				3D peritoneal model - apoptosis assay, submesothelial measurement
10	Menstrual period	33	32.8	2	Salpingectomy + hysterectomy	✓	✓				IF cells, HPF purity scoring,<br>3D peritoneal model - hydrogel matrices composition trial, apoptosis assay, submesothelial measurement
11	Proliferative	30	25.1	4	Tubal ligation	✓	✓				3D peritoneal model - donor 2 LDH assay and ELISA, apoptosis assay, IF<br>3D endometriosis model - IF, H&E, IHC
12	Secretory	23	30	0	Diagnostic laparoscopy			✓			3D endometriosis model - H&E, IHC, IF
13	HT	39	N/A	1	N/A	✓					3D peritoneal model - donor 1* LDH assay and ELISA, apoptosis assay, IF<br>3D endometriosis model - H&E, IHC, IF
14	Secretory	43	24.5	0	Menorrhagia			✓			IHC
15	Proliferative	27	N/A	2	N/A					✓	IF tissue
16	Secretory	26	41	0	Diagnostic laparoscopy			✓			Endometrial spheroid model - H&E, IHC
17	HT	60	21.7	2	N/A		✓				IF tissue
18	Secretory	37	20.9	0	Pelvic pain, menstrual bleeding	✓	✓				3D peritoneal model - IF

HT (on hormone treatment); N/A (data not available); PWF (peritoneal wash fluid); FTM (fallopian tube mesentery); EB (endometrial biopsy); UVF (uterovesical fold biopsy); ECT (ectopic lesion biopsy); IF (immunofluorescence staining); IHC (immunohistochemistry staining); LDH (lactate dehydrogenase); ELISA (enzyme-linked immunosorbent assay); H&E (Haematoxylin and Eosin). *Donor 1 peritoneal layer model used peritoneal fibroblast from patient no. 11.

Table S2. Quantitative histomorphometry measurement of the submesothelial thickness within 3D peritoneal layer model of the cell line and primary cell trial.

Cell type	Construct no.	Measurement no.	Submesothelial thickness (µm)	Average submesothelial thickness (µm)	Total average ± standard deviation (SD) (µm)
**Cell line (LP-9/NHDF)**	1	1	351	306.33	**313.97 ± 72.58**
		2	364.2		
		3	203.8		
	2	1	208.7	245.50	
		2	289.1		
		3	238.7		
	3	1	318.5	390.07	
		2	394.2		
		3	457.5		
**Primary cells (HPMCs/HPFs)**	1	1	486.7	459.97	**341.16 ± 112.08**
		2	523.7		
		3	369.5		
	2	1	239.7	237.30	
		2	238.4		
		3	233.8		
	3	1	407.2	326.20	
		2	282.8		
		3	288.6		

Table S3. Organoid expansion media used for endometrial epithelial organoid (EEO) expansion

Component	Manufacturer	Catalogue reference	Final concentration
DMEM/F12 Phenol-free	Gibco	21041025	Not applicable
N2 supplement	Gibco	17502048	1X
B27 supplement	Gibco	12587010	1X
Primocin	Invivogen	ant-pm-2	100 µg/mL
L-glutamine	Sigma-Aldrich	G7513	2 mM
Human noggin	Peprotech	120-10C	100 ng/mL
Human EGF	Peprotech	AF-100-15	50 ng/mL
Human HGF	Peprotech	100-39	50 ng/mL
Human FGF10	Peprotech	100-26	100 ng/mL
Human R-spondin-1	Peprotech	120-38	500 ng/mL
N-acetyl-L-cysteine	Sigma-Aldrich	A9165	1.25 mM
Nicotinamide	Sigma-Aldrich	128275000	10 nM
A83-01 ALK inhibitor	Biotechne	2939	500 nM

Table S4. List of antibodies used in this study

Protein	Manufacturer	Catalogue reference	Source, clonality, isotype	Assay	Dilution	Antigen retrieval method
Calponin	Millipore	ABT129	Rabbit polyclonal	IF	1 in 2,000	Not applicable
CD10	Santa Cruz Biotechnology	SC-46656	Mouse monoclonal IgG1	IHC	1 in 1,000	Citrate[1] pH 6.0, 2-3 h at 80°C
CD10	Abcam	ab309082	Rabbit monoclonal IgG	IF	1 in 100	Citrate[1] pH 6.0, 2-3 h at 80°C
CD31	Abcam	ab9498	Mouse monoclonal IgG1	IF	1 in 100	Not applicable
CD90	Abcam	ab181469	Mouse monoclonal IgG1	IF	1 in 100	Not applicable
Collagen IV	Developmental Studies Hybridoma Bank (DSHB), University of IOWA	M3F7	Mouse monoclonal IgG1	IF	1 in 10	Proteinase K[2], 20 min at RT
				IHC	1 in 25	
Cleaved Caspase-3	Cell Signaling technology	9664	Rabbit monoclonal IgG	IF	1 in 500	Citrate pH 6.0, 2-3 h at 80°C
Fibroblast Specific Protein (FSP)1/S100A4	Abcam	ab218512	Mouse monoclonal IgG1	IF	1 in 1,000	Citrate pH 6.0, 2-3 h at 80°C (tissue staining)
					1 in 20	Not applicable (cell staining)
				IHC	1 in 8,000	Citrate pH 6.0, 2-3 h at 80°C
Mesothelin	Biorbyt	orb1564633	Rabbit monoclonal IgG	IF	1 in 100	Citrate pH 6.0, 2-3 h at 80°C for tissue, not applicable for cell staining
pan-Cytokeratin	Sigma-Aldrich	c2562	Mouse monoclonal IgG1/IgG2A	IF	1 in 10,000	Citrate pH 6.0, 2-3 h at 80°C for tissue, not applicable for cell staining
				IHC	1 in 10,000	Citrate pH 6.0, 2-3 h at 80°C
Podoplanin	NeoBiotechnologies	10630-MSM1-P1ABX	Mouse monoclonal IgG1	IF	1 in 1,000	Citrate pH 6.0, 2-3 h at 80°C for tissue, not applicable for cell staining
				IHC	1 in 15,000	Citrate pH 6.0, 2-3 h at 80°C
Alpha-Smooth Muscle Actin (αSMA)	Sigma-Aldrich	a2547	Mouse monoclonal IgG2a	IF	1 in 100	Not applicable
				IHC	1 in 2,000	Citrate pH 6.0, 2-3 h at 80°C
TEM1	Abcam	ab204914	Rabbit monoclonal IgG	IF	1 in 250	Citrate pH 6.0, 2-3 h at 80°C for tissue, not applicable for cell staining
Vimentin	Abcam	ab8978	Mouse monoclonal IgG1	IF	1 in 800	Citrate pH 6.0, 2-3 h at 80°C
Vimentin	Abcam	ab45939	Rabbit polyclonal IgG	IF	1 in 400; 1 in 1,000	Not applicable
WT1	Abcam	ab89901	Rabbit monoclonal IgG	IF	1 in 100	Not applicable
Zonula Occludens-1 (ZO-1)	Invitrogen	40-2200	Rabbit polyclonal IgG	IF	1 in 100	Not applicable

[1]10 mM sodium citrate buffer; [2]20 µg/mL Proteinase K (Qiagen, 1014023).

Disease Models & Mechanisms • Supplementary information
